# Prevalence of *Plasmodium falciparum* Molecular Markers of Antimalarial Drug Resistance in a Residual Malaria Focus Area in Sabah, Malaysia

**DOI:** 10.1371/journal.pone.0165515

**Published:** 2016-10-27

**Authors:** Nor Azrina Norahmad, Mohd Ridzuan Mohd Abd Razak, Noor Rain Abdullah, Umi Rubiah Sastu, Mallika Imwong, Prem Kumar Muniandy, Muhammad Nor Farhan Saat, Amirrudin Muhammad, Jenarun Jelip, Moizin Tikuson, Norsalleh Yusof, Christina Rundi, Rose Nani Mudin, Ami Fazlin Syed Mohamed

**Affiliations:** 1 Herbal Medicine Research Center, Institute for Medical Research, Kuala Lumpur, Malaysia; 2 Department of Molecular Tropical Medicine and Genetics, Faculty of Tropical Medicine, Mahidol University, Bangkok, Thailand; 3 Sabah Vector Borne Disease Control Programme, Sabah State Health Department, Kota Kinabalu, Sabah, Malaysia; 4 Sabah District Health Office Kota Marudu, Kota Marudu, Sabah, Malaysia; 5 Vector Borne Disease Sector, Disease Control Division, Ministry of Health, Federal Government Administrative Centre, Putrajaya, Malaysia; Université Pierre et Marie Curie, FRANCE

## Abstract

Chloroquine (CQ) and fansidar (sulphadoxine-pyrimethamine, SP) were widely used for treatment of *Plasmodium falciparum* for several decades in Malaysia prior to the introduction of Artemisinin-based Combination Therapy (ACT) in 2008. Our previous study in Kalabakan, located in south-east coast of Sabah showed a high prevalence of resistance to CQ and SP, suggesting the use of the treatment may no longer be effective in the area. This study aimed to provide a baseline data of antimalarial drug resistant markers on *P*. *falciparum* isolates in Kota Marudu located in the north-east coast of Sabah. Mutations on genes associated with CQ (*pfcrt* and *pfmdr1*) and SP (*pfdhps* and *pfdhfr*) were assessed by PCR amplification and restriction fragment length polymorphism. Mutations on the kelch13 marker (K13) associated with artemisinin resistance were determined by DNA sequencing technique. The assessment of pfmdr1 copy number variation associated with mefloquine resistant was done by real-time PCR technique. A low prevalence (6.9%) was indicated for both *pfcrt* K76T and *pfmdr1* N86Y mutations. All *P*. *falciparum* isolates harboured the *pfdhps* A437G mutation. Prevalence of *pfdhfr* gene mutations, S108N and I164L, were 100% and 10.3%, respectively. Combining the different resistant markers, only two isolates were conferred to have CQ and SP treatment failure markers as they contained mutant alleles of *pfcrt* and *pfmdr1* together with quintuple *pfdhps/pfdhfr* mutation (combination of *pfdhps* A437G+A581G and *pfdhfr* C59R+S108N+I164L). All *P*. *falciparum* isolates carried single copy number of *pfmdr1* and wild type K13 marker. This study has demonstrated a low prevalence of CQ and SP resistance alleles in the study area. Continuous monitoring of antimalarial drug efficacy is warranted and the findings provide information for policy makers in ensuring a proper malaria control.

## Introduction

Malaria remains one of the major health concerns where approximately half of the world's population is at risk. World Health Organization (WHO) reports show more than 214 million malaria cases globally and about 438,000 lives lost in Africa, South East Asia and Eastern Mediterranean region in 2014 [1]. Malaysia has executed better strategies for improvement in prevention and control of malaria since the introduction of the Malaria Eradication Programme in 1960. In 2011, the Malaria Control Programme was restructured from control to elimination, and the Ministry of Health, Malaysia has begun to implement the National Strategic Plan for Malaria Elimination in 2011–2020 [2].

Malaysia is in the pre-elimination phase and continues to progress towards elimination, reporting 3923 confirmed malaria cases in 2014 [1]. The incidence rate of malaria has declined from 16.1 per 100,000 populations in 2012 to 13.0 per 100,000 populations in 2014. Even though malaria control activities have significantly reduced malaria incidence in Malaysia, the disease still remain as main public health problem in the less developed areas of the country especially in Sabah. About forty-two percent of the malaria cases in Malaysia were reported from Sabah in the year 2013 [3].

In Malaysia, development of parasite resistance toward antimalarial drugs has led to increasing difficulties for sufficient malaria disease management and elimination. The widespread of resistance towards chloroquine (CQ) and sulphadoxine-pyrimethamine (SP) [4–9] has led Malaysia to change their antimalarial treatment policies to Riamet, a combination drug of artemether and lumefantrine (AL) for non-complicated *P*. *falciparum* malaria; while doxycycline are be given orally together with intravenous artesunate for complicated falciparum malaria treatment [2, 10].

Single nucleotide polymorphisms (SNPs) in *P*. *falciparum* CQ transporter (*pfcrt*) gene (K76T) and *P*. *falciparum* multidrug-resistance 1 (*pfmdr1*) gene (N86Y) have been associated with CQ resistance [11]. Additionally, several *in vitro* studies have demonstrated the association of high *pfmdr1* copy number with lower parasites susceptibility to mefloquine and halofantrine [12, 13].

Resistance to SP drug combination has been shown to occur due to the alteration in the amino acid sequences of the *P*. *falciparum* dihydrofolate reductase (*pfdhfr*) [14] and *P*. *falciparum* dihydropteroate synthase (*pfdhps*) genes [15]. Specific changes of amino acid serine to asparagine at codon 108 (S108N) or isoleucine to leucine at codon 164 (I164L) on *pfdhfr* gene have been identified as the key determinants in the evolution of pyrimethamine (PYR) resistant *in vitro* [11]. The severity of pyrimethamine resistance often enhances by additional 51I and/or 59R mutation. Meanwhile, A437G and A581G point mutations on *pfdhps* gene confer resistance to sulphadoxine (SDX) *in vitro* enhanced by the presence of S436A, K540E and A613S [11]. Multiple mutation combinations of both *pfdhps* and *pfdhfr* were responsible in varying the degrees of SP resistance [11, 16, 17].

Resistance to artemisinin based combination therapies (ACT) has been observed in western Cambodia, Thailand, Vietnam, and Myanmar [18–21]. A previous study has identified mutations in the propeller domain of a kelch gene on chromosome 13 (PF3D7_1343700, K13 gene) as candidate molecular markers of ART resistance [22]. The prevalence of K-13 propeller region mutant alleles have been associated with parasite delayed clearance [22] played by Y493H, C580Y, M476I, R539T and I543T mutations [23].

To achieve malaria elimination status, wide coverage of molecular data on antimalarial drug resistance in Malaysia is needed for proper implementation of antimalarial drug treatment policy. Therefore, the aim of this study is to assess the prevalence of point mutations in the genes associated with CQ and SP resistance such as *pfcrt* (codon 76), *pfmdr1* (codon 86), *pfdhfr* (codons 16, 51, 59, 108 and 164) and *pfdhps* (codons 437, 540, 581) on *P*. *falciparum* isolated in Kota Marudu, Sabah. In addition, we have also assessed the status of K-13 propeller polymorphisms and high *pfmdr1* copy number variation which have been associated with artemisinin and mefloquine resistance, respectively. The data from this study could also contribute to a baseline information on distribution of antimalarial drug resistance particularly in Sabah prior to malaria elimination.

## Materials and Methods

### Study Site

Kota Marudu is one of the districts in Kudat division of Sabah with approximately 19.17 square kilometres of land. The population in Kota Marudu as in 2009 is approximately 72,900 with the average population of five per household [24]. In the first quarter of 2011, malaria cases in Kota Marudu contributed 10% of total malaria cases in Sabah. The majority of malaria cases in this area was majorly caused by *P*. *falciparum* infection followed by *P*. *vivax* and *P*. *malariae*. The district also falls under the list of high malaria endemic area in Sabah with total malaria endemicity of 10,000–50,000 [25].

### Ethics approval and consent to participate

The study protocol was reviewed and approved by the Research Review Committee (RRC) of the Institute for Medical Research (IMR) and the Medical Research Ethics Committee (MREC), Ministry of Health Malaysia. All individuals were given a detailed explanation of the study procedures. Written informed consent was obtained from adult individuals or from parents or guardians of children under the age of 18 years.

### Sample Collection

Cross-sectional community malaria screening surveys were conducted in malaria endemic areas of Kota Marudu as suggested by the Sabah State Health Department and Kota Marudu District Health Office in 2011 and 2014. Each screening survey was conducted in different areas. All blood samples from 4049 individuals (symptomatic and asymptomatic) were randomly collected by active case detection in more than 50 sites in deep forested areas or villages in Kota Marudu such as Sonsogun Mogis, Sonsogun Magandai, Pintasan Darat, Mampakad, Pinatau, Lotong, Launa, Lembiding, Linkabungan, Sunsui and Gana. The individuals were recruited at meeting points in each villages. House to house screening survey was also conducted. In addition, 21 *P*. *falciparum* infected samples from Kalabakan, Sabah were included in this study but limited to K13 propeller domain mutations and pfmdr1 copy number variation assessment. Blood film for malaria parasite technique (BFMP) was also prepared to confirm the infection. Malaria infected individuals were advised and transported to the nearest public hospitals for treatment.

Blood sample was obtained by finger prick and malaria infection was diagnosed using rapid diagnostic test kit (Paramax-3™, Zephyr Biomedicals, India). Approximately 100 μl of malaria infected bloods were spotted onto 3MM^®^ Whatman (Brentford, United Kingdom) filter paper. The dried filter papers were labelled and transferred into individual plastic bags before being transported to the IMR in Kuala Lumpur, Malaysia. The blood-spotted filter papers were stored at room temperature in a dessicator containing silica gel until further processing.

### DNA Extraction and Species Identification

Malaria parasite genomic DNA was extracted from filter papers using QIAmp DNA Mini Kit (QIAGEN, Germany) according to the manufacturer's instructions. A similar protocol was used to extract genomic DNA from the laboratory clone strains of *P*. *falciparum* (3D7, K1, T9.96 and W2) for PCR controls. *Plasmodium* species identification was also performed on malaria-infected samples by PCR as previously described [11, 26].

### Molecular Analysis of *Pfcrt*, *Pfmdr1*, *Pfdhps*, *Pfdhfr* and K13 Propeller Domain

All PCRs were performed by using an Eppendorf Mastercycler Gradient (Eppendorf, Germany). The DNA from established laboratory strains of *P*. *falciparum* served as controls for PCR and enzyme digestions. Water was used to replace the DNA template in the PCR reaction for negative control. All restriction enzymes were brought from New England Biolabs (Beverly, Massachusetts, USA). The PCR products were analyzed by using the Agilent 2100 Bioanalyzer and the Agilent DNA 1000 Kit (Agilent Technologies, Molecular Probes Inc, USA). The PCR products were cleaned using QIAquick PCR Purification Kit (QIAGEN) before sending for sequencing. The DNA sequences were analyzed by using DNASTAR (Lasergene) and by using Molecular Evolutionary Genetics Analysis (MEGA) version 6.0 software.

The *pfcrt* K76T mutation analysis was performed as described by Djimde et al. [27] with minor adjustments to the concentrations of the reagents used for the PCR reaction [6]. The N86Y mutation in *pfmdr1* gene was performed as described elsewhere [28].

Detection of *pfdhps* mutations at residues 437, 540, and 581 and *pfdhfr* mutations at residues 6, 51, 59, 108 and 164 were done as previously described [29] with some modification to DNA and primer concentrations [30]. The secondary PCR products containing the target region were subjected to RLFP for the detection of mutations at the various sites. The enzyme digestions were carried out according to published methods by Duraisingh et al. [29] and Jelinek et al. [31] for both *pfdhps* and *pfdhfr* genes, respectively.

The K13-propeller domain was amplified by using nested PCR as described by Ariey et al. [22]. The nested PCR products were evaluated by using Bioanalyzer and sent for sequencing. Sequences were assembled and manually edited by using DNASTAR (WI, USA). DNA sequences alignments were performed with the K13 sequence of the 3D7 clone (PF3D7_1343700) retrieved from PlasmoDB as reference sequence by using Molecular Evolutionary Genetics Analysis (MEGA) version 6.0 software.

### Quantitation of *Pfmdr1* Copy Number using Real-time Quantitative PCR

Real time PCR was performed with Rotor-Gene^®^ Q (QIAGEN). Amplification reactions were done in triplicate by multiplex PCR combining both *pfmdr1* and β-tubulin primers with probes as previously described [32]. The reagents used for each sample were 1X QuantiTect Multiplex (2X, NoROX), 400 nM of each forward and reverse *pfmdr1* primer, 200 nM of *pfmdr1* probe, 400 nM of each forward and reverse β-tubulin primer, 200 nM of β-tubulin probe, 2.5 μl of template DNA and sterile water in total volume of 10 μl. β-tubulin served as an internal standard for the amout of sample added to the reactions. *Pfmdr1* copy number was calculated by the following formula: Copy number = 2^-ΔΔC^_t_ with ΔΔC_t_ denoting the difference between ΔC_t_ of the unknown sample and ΔC_t_ of the reference sample. The Efficiency (E) of the β-tubulin was assumed to be 2. The 3D7 (1 copy number) and IC (2 copies number) laboratory clone was used as the reference DNA sample respectively.

## Results

### Total samples

A total number of 4049 individuals were screened for malaria infection. Eighty-seven (2.15%, 87/4049) were positive for malaria infection and twenty-nine (33.33%, 29/87 malaria infected individuals) were infected with *P*. *falciparum*. All isolates were successfully genotyped by PCR-RFLP for drug resistant genes *pfcrt*, *pfmdr1*, *pfdhps* and *pfdhfr*. We evaluated mutant alleles in various locus: *pfcrt* (K76T), *pfmdr1* (N86Y), *pfdhps* (A437G, K540E and A581G) and *pfdhfr* (A16V, N51I, C59R, S108T/N and I164L). In addition, pfmdr1 copy number variation and K13 propeller region point mutations were also determined in this study. Besides 29 Kota Marudu samples, an additional of 21 Kalabakan *P*. *falciparum* infected samples collected between 2008 to 2009 were included for pfmdr1 copy number variation and K13 propeller region mutations analysis. The *P*. *falciparum* isolates from Kalabakan have been previously analyzed for CQ and SP resistances [5, 6].

### Pfcrt and pfmdr1

As shown in Table 1, the frequency of the pure mutant allele for both *pfcrt* K76T and *pfmdr1* N86Y were low with 6.9% (2/29) prevalence, respectively. Only one mutant genotype for *pfcrt* and *pfmdr1* gene was identified among the isolates in which, two samples harboured both mutant alleles for *pfcrt* and *pfmdr1* gene (Table 1).

**Table 1:** Frequency of wild type and mutant alleles of *pfcrt*, *pfmdr1*, *pfdhfr* and *pfdhps* in *P*. *falciparum* isolates from Kota Marudu, Sabah.

Drug resistant	Chloroquine		Sulfadoxine			Pyrimethamine				
Genes	*Pfcrt*	*Pfmdr1*	*Pfdhps*			*Pfdhfr*				
Mutant Codon	K76T	N86Y	A437G	K540E	A581G	A16V	N51I	C59R	S108N	I164L
Frequency (%)	**6.9**	**6.9**	**100**	**0**	**6.9**	**0**	**0**	**100**	**100**	**10.3**
No. of mutant samples	**2/29**	**2/29**	**29/29**	**0/29**	**2/29**	**0/29**	**0/29**	**29/29**	**29/29**	**3/29**

*Pfcrt*, *P*. *falciparum* chloroquine transporter gene; *pfmdr1*, *P*. *falciparum* multidrug resistant gene; *pfdhfr*, *P*. *falciparum* dihydropteroate synthase, *pfdhps*, *P*. *falciparum* dihydrofolate reductase. Amino acids: A, alanine, C, cysteine, E, glutamic acid, G, glycine, I, isoleucine, K, lysine, L, leucine, N, asparagine, R, arginine, S, serine, T, threonine, V, valine.

### Pfdhps and pfdhfr

For the *pfdhps*, all isolates (N = 29) carried the essential A437G mutant allele. None of the isolates carrying K540E mutant allele. Two isolates (6.9%, 2/29) carried mutant alleles for A581G (Table 1). Out of 29 samples, only 2 isolates were found to have double mutations in *pfdhps* specifically at A437G + A581G (6.9%, 2/29).

For the *pfdhfr*, 3 isolates (10.3%, 3/29) harboured a mutation at codon I164L. Double mutations at codon C59R and S108N was found in all samples while mutations at codon A16V and N51I were absent (Table 1). Three isolates harboured triple mutations at C59R + S108N + I164L (10.3%, 3/29).

Combining the *pfdhps* and *pfdhfr* mutations, 3 genotypes were identified. The most common combinations were triple mutation of A437G (*Pfdhps*) + C59R + S108N (*Pfdhfr*) (89.7%, 26/29) (Table 2). There were two isolates (6.9%, 2/29 isolates) with quintuple mutation of A437G + A581G (*Pfdhps*) + C59R + S108N + I164L (*Pfdhfr*) followed by 1 isolate (3.4%, 1/29) with quadruple mutation of A437G (*Pfdhps*) + C59R + S108N + I164L (*Pfdhfr*) (Table 2).

**Table 2:** Frequency of combined *pfdhfr* /*pfdhps* mutants in isolates from Kota Marudu.

*Pfdhps codons*			*Pfdhfr codons*					Frequency of mutant genotype		
437	540	581	16	51	59	108	164	pfdhfr/ pfdhps combination	% of mutant genotype	No. of mutants samples
G	L	A	A	N	***R***	***N***	I	1 pfdhps/ 2 pfdhfr	89.7	26/29
G	L	A	A	N	***R***	***N***	***L***	1 pfdhps/ 3 pfdhfr	3.4	1/29
G	L	***G***	A	N	***R***	***N***	***L***	2 pfdhps/ 3 pfdhfr	6.9	2/29

*Pfcrt*, *P*. *falciparum* chloroquine transporter gene; *pfmdr1*, *P*. *falciparum* multidrug resistant gene; *pfdhfr*, *P*. *falciparum* dihydropteroate synthase, *pfdhps*, *P*. *falciparum* dihydrofolate reductase. Amino acids: A, alanine, C, cysteine, E, glutamic acid, G, glycine, I, isoleucine, K, lysine, L, leucine, N, asparagine, R, arginine, S, serine, T, threonine, V, valine. Mutation is represented by amino acid in italic bold.

### K13 propeller region and *pfmdr1* copy number

The propeller region of K13 gene was successfully sequenced for *P*. *falciparum* isolates from Kota Marudu (N = 29) and Kalabakan (N = 21). The sequencing results of these isolates revealed no polymorphisms were detected in all 17 locations in K13 propeller region which confer resistance to ACT (S1 Fig).

For *pfmdr1* copy number variation analysis, only 22 Kota Marudu and 16 Kalabakan isolates were successfully amplified by RT-PCR. Both *P*. *falciparum* isolates from Kota Marudu (N = 22) and Kalabakan (N = 16) were found to carry one copy number of *pfmdr1* gene (S1 Table).

## Discussion

**Fig 1:**
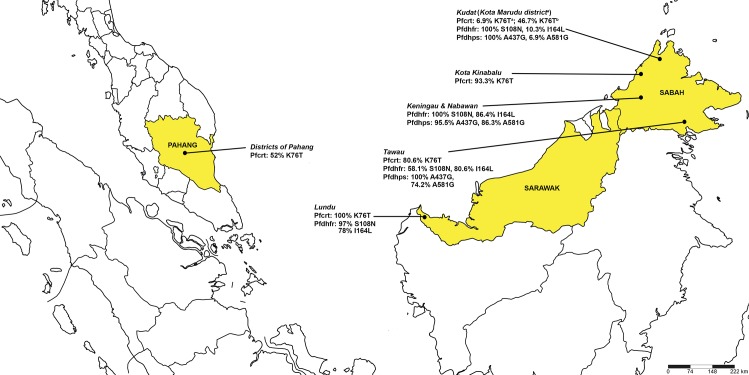
Distribution of essential mutations for CQ, PYR and SDX resistance in Malaysia. The map shows the distribution of common SNPs for *P*. *falciparum* CQ, PYR and SDX resistant previously reported in Pahang [9], Lundu, Sarawak [8] and Sabah: Kalabakan (Tawau Division) [6, 30], Keningau & Nabawan (Interior Division) [39], Kota Kinabalu (West Coast Division) [35], ^a^Kota Marudu—present study, ^b^Kudat [35] (Kudat Division). This map was generated by using SimpleMappr online software [40]. *Pfcrt*, *Plasmodium falciparum* chloroquine resistant gene; *Pfdhfr*, *Plasmodium falciparum* dihydrofolate reductase gene; *Pfdhps*, *Plasmodium falciparum* dihydropteroate synthase gene; CQ, chloroquine; SDX, sulphadoxine; PYR, pyrimethamine. Amino acids: A, alanine; G, glycine; I, isoleucine; K, lysine; L, leucine N, asparagine; S, serine; T, threonine.

Our results demonstrated a low prevalence (6.9%) of *pfcrt* K76T mutation associated with resistance to CQ in *P*. *falciparum* isolated from Kota Marudu, Kudat Division of Sabah (Fig 1 and Table 3). Moreover, the same low percentage of mutation was detected for *pfmdr1* N86Y, which is known to contribute to CQ resistance [33, 34]. Another study conducted in 2012 has detected 46.7% (7/15) of K76T mutation on clinical *P*. *falciparum* isolates in Kudat, Sabah (Fig 1 and Table 3). In contrast, a high prevalence rate of *pfcrt* K76T mutation was previously observed in Kalabakan and Kota Kinabalu, located in Tawau division and West Coast Division of Sabah, respectively (Fig 1 and Table 3) [6, 35]. A hundred percent prevalence of K76T mutation was reported in Lundu, Sarawak in a study conducted in 1999 and 2000 (Fig 1 and Table 3) [8]. A previous study by Atroosh et al.[9] have reported high prevalence rate of *pfcrt* K76T mutation in *P*. *falciparum* isolates in the Peninsular Malaysia (West Malaysia) area such as Pahang (Fig 1 and Table 3). The neighbouring countries including Thailand, Indonesia and Philippine [36–38] have also shown a high prevalence of the same mutation.

**Table 3:** Prevalence of CQ, PYR and SDX resistant marker genotypes in Malaysia reported from year 2003 to 2014.

Sampling site	Year of sampling	Sampling method	Resistance	Genes	Codon mutations	Frequency (%)	Total sample	References
Lundu, Sarawak	1999 and 2000	PCD	CQ	*pfcrt*	**K76T**	100.0	47/47	Cox-Singh et al. 2003 [8]
			PYR	*pfdhfr*	C59R/**S108N**	19.0	6/47	
			PYR	*pfdhfr*	C59R/**S108N**/**I164L**	78.0	25/47	
Pahang districts	2010–2011	ACD/PCD	CQ	*pfcrt*	**K76T**	52.0	39/75	Atroosh et al. 2012 [9]
Kalabakan (Tawau), Sabah	2008–2009	ACD	CQ	*pfcrt*	**K76T**	80.6	25/31	Norahmad et al. 2011 [6]
			PYR	*pfdhfr*	C59R/**S108N**	3.2	1/31	Abdullah et al. 2013 [5]
					C59R/**I164L**	22.6	7/31	
					C59R/**S108N**/**I164L**	51.6	16/31	
					A16V/C59R/**I164L**	3.2	1/31	
					A16V/C59R/**S108N**/**I164L**	3.2	1/31	
			SDX	*pfdhps*	**A437G**/**A581G**	74.2	23/31	
Keningau/Nabawan, Sabah	2010	PCD	PYR	*pfdhfr*	C59R/**S108N**/**I164L**	86.4	19/22	Lau et al. 2013 [39]
					N51I/C59R/**S108N**	4.5	1/22	
					C59R/**S108N**	9.1	2/22	
			SDX	*pfdhps*	**A437G**/K540T/**A581G**	72.7	16/22	
					**A437G**	9.1	2/22	
					**A437G**/**A581G**	13.6	3/22	
Kota Kinabalu, Sabah	2012	PCD	CQ	*pfcrt*	**K76T**	93.3	14/15	Tan et al. 2014 [35]
Keningau, Sabah	2012	PCD	CQ	*pfcrt*	**K76T**	100	1/1	
Kudat, Sabah	2012	PCD	CQ	*pfcrt*	**K76T**	46.7	7/15	
Kota Marudu (Kudat), Sabah	2011 and 2014	ACD	CQ	*pfcrt*	**K76T**	6.9	2/29	This study
			PYR	*pfdhfr*	C59R**/S108N**	89.7	26/29	
					C59R/**S108N**/**I164L**	10.3	3/29	
			SDX	*pfdhps*	**A437G**	93.1	27/29	
					**A437G**/**A581G**	6.9	2/29	

ACD, active case detection; PCD, passive case detection; *Pfcrt*, *Plasmodium falciparum* chloroquine resistant gene; *Pfdhfr*, *Plasmodium falciparum* dihydrofolate reductase gene; *Pfdhps*, *Plasmodium falciparum* dihydropteroate synthase gene; CQ, chloroquine; SDX, sulphadoxine; PYR, pyrimethamine. Amino acids: A, alanine; C, cysteine; G, glycine; I, isoleucine; K, lysine; L, leucine; N, asparagine; R, arginine; S, serine; T, threonine; V, valine. Bold fonts indicates strong determinant of resistance for each antimalarial drug.

Mutations on *pfdhps* and *pfdhfr* genes associated with SP resistance have been reported in most part of malaria endemic areas in Sabah and Sarawak (Fig 1 and Table 3) [5, 8, 41] and the neighbouring South Kalimantan, Indonesia [42]. Combination of triple *pfdhfr* mutation and double *pfdhps* mutation (quintuple mutant) have been associated with SP treatment failure [16]. Based on the previous study carried out by Cox-Singh et al. [8] in Lundu, Sarawak, triple *pfdhfr* mutation (C59R/S108N/I164L) could also lead to SP treatment failure. However, the status of *pfdhps* mutation was not reported in the latter study. Our study showed that all *P*. *falciparum* isolates were harbouring essential mutant alleles in *pfdhps* (A437G and A581G) and *pfdhfr* genes (S108N and I164L) which confer to SDX and PYR resistances, respectively [11]. Triple mutation involving a combination of 1 mutant allele of *pfdhps* and 2 mutant alleles of *pfdhfr* (A437G/C59R/S108N) were predominant followed by low prevalence of quadruple (A437G/C59R/S108N/I164L) and quintuple (A437G/A581G/C59R/S108N/I164L) mutations combinations (Table 2). These combinations have also been observed in Keningau and Nabawan district in the interior of Sabah [41] and Kalabakan in the south-eastern coast of Sabah [30]. Although the prevalence of quadruple and quintuple combining mutations of *pfdhps* and *pfdhfr* were low, double mutations on *pfdhfr* alone have been associated with longer parasite clearance time and higher gametocytemia, the presence of gametocytes responsible for transmission [17].

An increased copy number of *pfmdr1* gene was associated with *in vitro* and *in vivo* resistance towards mefloquine (MQ) and AL antimalarial drugs [32, 43]. It has been reported that due to frequent usage of MQ monotherapy in some parts of Thailand and Cambodia, the prevalence of *P*. *falciparum* isolates with a *pfmdr1* copy number greater than 1 was found to be high [32]. Currently, the ASMQ fixed-dose combination is recommended as alternative treatment to AL in treating uncomplicated *P*. *falciparum* malaria in Malaysia [2]. The absence of increased *pfmdr1* copy number suggests the efficacy of MQ in the study area.

*P*. *falciparum* resistance towards artemisinin is a major setback for malaria control in Southeast Asia. The neighbouring countries such as Thailand, Myanmar and Cambodia have actively reported the prevalence of C580Y which was the marker for slow-clearing *P*. *falciparum* in malaria patients treated with artemisinin and ACT [19, 20, 22]. Although the delayed parasite clearance after treatment with ACT has not yet been reported in Malaysia, characterizing the diversity of this gene is important to assess the potential for ACT drug resistance and to provide a baseline for future surveillance. Therefore, we sequenced the K13 propeller domain in *P*. *falciparum* isolates from Kota Marudu and Kalabakan. As this treatment has just been introduced in Malaysia, the absence of K13 propeller mutations in *P*. *falciparum* isolates were expected in the study area.

As Malaysia has been listed as one of the pre-elimination phase countries, a fragmented population structure of the *P*. *falciparum* populations is expected [44]. For example, the frequency of CQ resistant parasite in Kota Marudu were extremely low as compared to other areas such as Kalabakan. This suggested that the spread of resistant *P*. *falciparum* genotype in the region was contained within the malaria focus area. In addition, the genetic differentiation analysis has assured the large variation of genetic pattern between the *P*. *falciparum* populations in the areas of Sabah [45, 46]. Another possible explanation to this is the different sampling period between present study and previous studies conducted in Kalabakan [6, 30] and Pahang [9] areas where samples were collected before the implementation of ACT in the country. The effect of antimalarial drug interventions such as the use of AL in 2008 might have caused the extinction of CQ resistant *P*. *falciparum* lineages. The selection of the strongest *P*. *falciparum* population after ACT interventions had been demonstrated in Ghana and South American [47, 48].

As a developing area, malaria cases in Kota Marudu have become more confined to the rural population living in less accessible, hilly, forested hinterland, and to areas with inadequate transportation and communication facilities. These situations have made the ACD malaria screening in the study areas more challenging. The limitation of this study is the small number of sample (N = 29) collected in year 2011 and 2014. However, the study has randomly screened more than 4000 individuals living in the foci areas by ACD and it reflects the declining of malaria cases reported in Malaysia during the period of the study. Continuous molecular surveillance of antimalarial drug resistant markers is recommended to track the emergence and spread of *P*. *falciparum* mutation towards CQ, SP and ART resistance. This effort is also crucial to ensure the efficacy of malaria treatment and control programs, particularly in East Malaysia; Sabah and Sarawak.

## Conclusion

This was the first molecular study carried out in this geographical area focusing on mutations of *pfcrt*, *pfmdr1*, *pfdhps* and *pfdhfr* genes that were strongly associated to CQ and SP resistance. This study showed low prevalence of resistance markers to CQ and SP that dramatically contrasted with the pattern observed with our previous study in Kalabakan, where higher *pfcrt* mutant allele and quintuple *pfdhfr*/*pfdhps* mutation were observed. Additionally, absence of increased *pfmdr1* copy number and K-13 propeller domain mutations are expected due to limited usage of MQ and ACT in the study area.

## Supporting Information

S1 Fig Sequencing alignment of K13 propeller region at amino acid position 435–690. (PDF)

S1 Table Copy number of *pfmdr1* on the *P*. *falciparum* isolates. (PDF)
