# Supplementary material for: Prevalence of Plasmodium falciparum Molecular Markers of Antimalarial Drug Resistance in a Residual Malaria Focus Area in Sabah, Malaysia
Source: PLoS One. 2016 Oct 27;11(10):e0165515. doi: 10.1371/journal.pone.0165515 (PMC5082862; doi:10.1371/journal.pone.0165515)
Supplement: S1 Fig — (PDF) [file pone.0165515.s001.pdf]

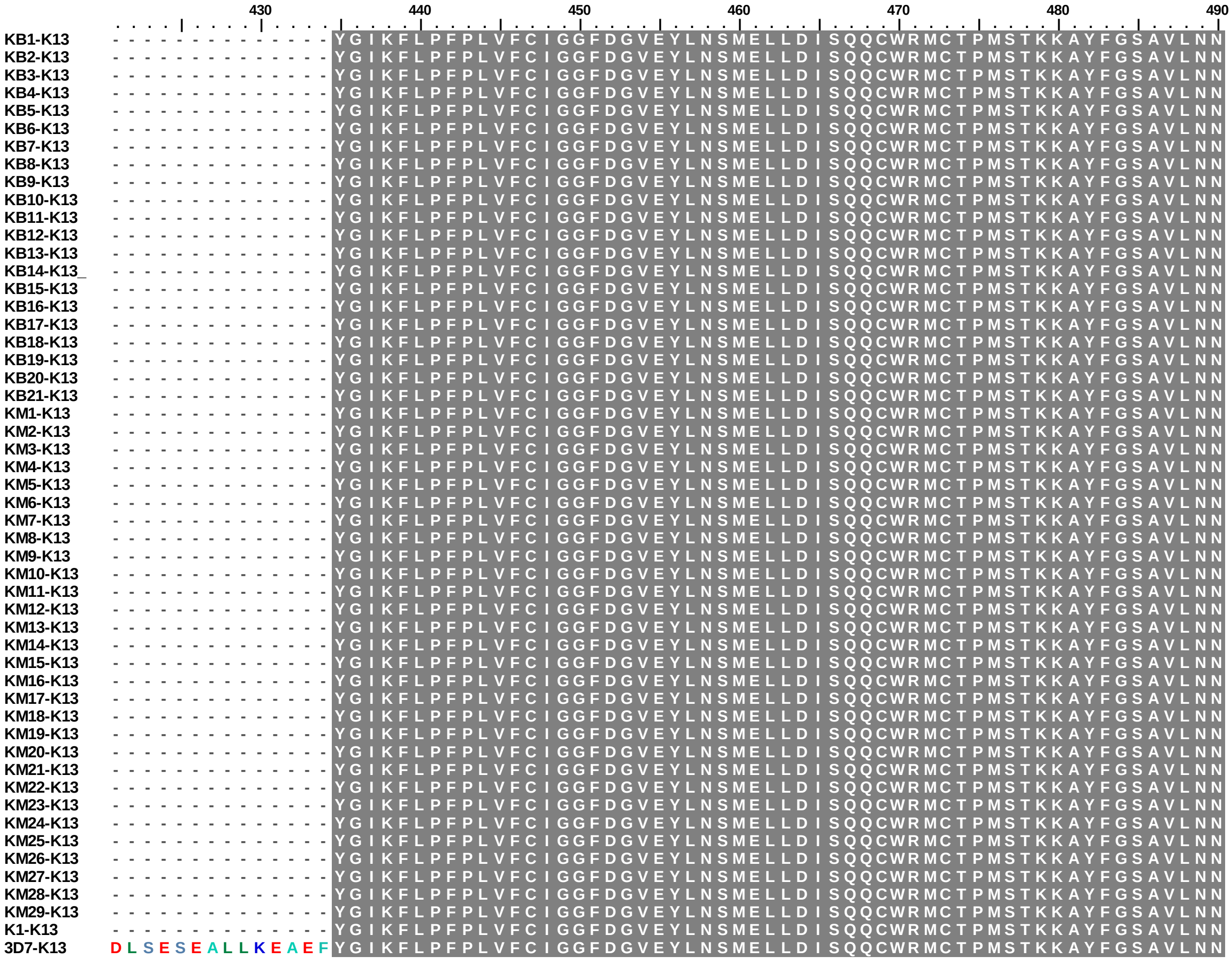

S1 Fig. Sequencing alignment of K13 propeller region at amino acid position 435-690. The *P. falciparum* 3D7 and K1 strains are the artemisinin sensitive control. KM, Kota Marudu isolate and KB, Kalabakan isolate.

	500 · 510 · 520 · 530 · 540 · 550 · 560
KB1-K13	FLYVFGGNNYDYKALFETEVYDRLRDVWYVSSNLNIPRRNNCGVTSNGRIYCIGGYDGSSIIPNVEAYDH
KB2-K13	FLYVFGGNNYDYKALFETEVYDRLRDVWYVSSNLNIPRRNNCGVTSNGRIYCIGGYDGSSIIPNVEAYDH
KB3-K13	FLYVFGGNNYDYKALFETEVYDRLRDVWYVSSNLNIPRRNNCGVTSNGRIYCIGGYDGSSIIPNVEAYDH
KB4-K13	FLYVFGGNNYDYKALFETEVYDRLRDVWYVSSNLNIPRRNNCGVTSNGRIYCIGGYDGSSIIPNVEAYDH
KB5-K13	FLYVFGGNNYDYKALFETEVYDRLRDVWYVSSNLNIPRRNNCGVTSNGRIYCIGGYDGSSIIPNVEAYDH
KB6-K13	FLYVFGGNNYDYKALFETEVYDRLRDVWYVSSNLNIPRRNNCGVTSNGRIYCIGGYDGSSIIPNVEAYDH
KB7-K13	FLYVFGGNNYDYKALFETEVYDRLRDVWYVSSNLNIPRRNNCGVTSNGRIYCIGGYDGSSIIPNVEAYDH
KB8-K13	FLYVFGGNNYDYKALFETEVYDRLRDVWYVSSNLNIPRRNNCGVTSNGRIYCIGGYDGSSIIPNVEAYDH
KB9-K13	FLYVFGGNNYDYKALFETEVYDRLRDVWYVSSNLNIPRRNNCGVTSNGRIYCIGGYDGSSIIPNVEAYDH
KB10-K13	FLYVFGGNNYDYKALFETEVYDRLRDVWYVSSNLNIPRRNNCGVTSNGRIYCIGGYDGSSIIPNVEAYDH
KB11-K13	FLYVFGGNNYDYKALFETEVYDRLRDVWYVSSNLNIPRRNNCGVTSNGRIYCIGGYDGSSIIPNVEAYDH
KB12-K13	FLYVFGGNNYDYKALFETEVYDRLRDVWYVSSNLNIPRRNNCGVTSNGRIYCIGGYDGSSIIPNVEAYDH
KB13-K13	FLYVFGGNNYDYKALFETEVYDRLRDVWYVSSNLNIPRRNNCGVTSNGRIYCIGGYDGSSIIPNVEAYDH
KB14-K13_	FLYVFGGNNYDYKALFETEVYDRLRDVWYVSSNLNIPRRNNCGVTSNGRIYCIGGYDGSSIIPNVEAYDH
KB15-K13	FLYVFGGNNYDYKALFETEVYDRLRDVWYVSSNLNIPRRNNCGVTSNGRIYCIGGYDGSSIIPNVEAYDH
KB16-K13	FLYVFGGNNYDYKALFETEVYDRLRDVWYVSSNLNIPRRNNCGVTSNGRIYCIGGYDGSSIIPNVEAYDH
KB17-K13	FLYVFGGNNYDYKALFETEVYDRLRDVWYVSSNLNIPRRNNCGVTSNGRIYCIGGYDGSSIIPNVEAYDH
KB18-K13	FLYVFGGNNYDYKALFETEVYDRLRDVWYVSSNLNIPRRNNCGVTSNGRIYCIGGYDGSSIIPNVEAYDH
KB19-K13	FLYVFGGNNYDYKALFETEVYDRLRDVWYVSSNLNIPRRNNCGVTSNGRIYCIGGYDGSSIIPNVEAYDH
KB20-K13	FLYVFGGNNYDYKALFETEVYDRLRDVWYVSSNLNIPRRNNCGVTSNGRIYCIGGYDGSSIIPNVEAYDH
KB21-K13	FLYVFGGNNYDYKALFETEVYDRLRDVWYVSSNLNIPRRNNCGVTSNGRIYCIGGYDGSSIIPNVEAYDH
KM1-K13	FLYVFGGNNYDYKALFETEVYDRLRDVWYVSSNLNIPRRNNCGVTSNGRIYCIGGYDGSSIIPNVEAYDH
KM2-K13	FLYVFGGNNYDYKALFETEVYDRLRDVWYVSSNLNIPRRNNCGVTSNGRIYCIGGYDGSSIIPNVEAYDH
KM3-K13	FLYVFGGNNYDYKALFETEVYDRLRDVWYVSSNLNIPRRNNCGVTSNGRIYCIGGYDGSSIIPNVEAYDH
KM4-K13	FLYVFGGNNYDYKALFETEVYDRLRDVWYVSSNLNIPRRNNCGVTSNGRIYCIGGYDGSSIIPNVEAYDH
KM5-K13	FLYVFGGNNYDYKALFETEVYDRLRDVWYVSSNLNIPRRNNCGVTSNGRIYCIGGYDGSSIIPNVEAYDH
KM6-K13	FLYVFGGNNYDYKALFETEVYDRLRDVWYVSSNLNIPRRNNCGVTSNGRIYCIGGYDGSSIIPNVEAYDH
KM7-K13	FLYVFGGNNYDYKALFETEVYDRLRDVWYVSSNLNIPRRNNCGVTSNGRIYCIGGYDGSSIIPNVEAYDH
KM8-K13	FLYVFGGNNYDYKALFETEVYDRLRDVWYVSSNLNIPRRNNCGVTSNGRIYCIGGYDGSSIIPNVEAYDH
KM9-K13	FLYVFGGNNYDYKALFETEVYDRLRDVWYVSSNLNIPRRNNCGVTSNGRIYCIGGYDGSSIIPNVEAYDH
KM10-K13	FLYVFGGNNYDYKALFETEVYDRLRDVWYVSSNLNIPRRNNCGVTSNGRIYCIGGYDGSSIIPNVEAYDH
KM11-K13	FLYVFGGNNYDYKALFETEVYDRLRDVWYVSSNLNIPRRNNCGVTSNGRIYCIGGYDGSSIIPNVEAYDH
KM12-K13	FLYVFGGNNYDYKALFETEVYDRLRDVWYVSSNLNIPRRNNCGVTSNGRIYCIGGYDGSSIIPNVEAYDH
KM13-K13	FLYVFGGNNYDYKALFETEVYDRLRDVWYVSSNLNIPRRNNCGVTSNGRIYCIGGYDGSSIIPNVEAYDH
KM14-K13	FLYVFGGNNYDYKALFETEVYDRLRDVWYVSSNLNIPRRNNCGVTSNGRIYCIGGYDGSSIIPNVEAYDH
KM15-K13	FLYVFGGNNYDYKALFETEVYDRLRDVWYVSSNLNIPRRNNCGVTSNGRIYCIGGYDGSSIIPNVEAYDH
KM16-K13	FLYVFGGNNYDYKALFETEVYDRLRDVWYVSSNLNIPRRNNCGVTSNGRIYCIGGYDGSSIIPNVEAYDH
KM17-K13	FLYVFGGNNYDYKALFETEVYDRLRDVWYVSSNLNIPRRNNCGVTSNGRIYCIGGYDGSSIIPNVEAYDH
KM18-K13	FLYVFGGNNYDYKALFETEVYDRLRDVWYVSSNLNIPRRNNCGVTSNGRIYCIGGYDGSSIIPNVEAYDH
KM19-K13	FLYVFGGNNYDYKALFETEVYDRLRDVWYVSSNLNIPRRNNCGVTSNGRIYCIGGYDGSSIIPNVEAYDH
KM20-K13	FLYVFGGNNYDYKALFETEVYDRLRDVWYVSSNLNIPRRNNCGVTSNGRIYCIGGYDGSSIIPNVEAYDH
KM21-K13	FLYVFGGNNYDYKALFETEVYDRLRDVWYVSSNLNIPRRNNCGVTSNGRIYCIGGYDGSSIIPNVEAYDH
KM22-K13	FLYVFGGNNYDYKALFETEVYDRLRDVWYVSSNLNIPRRNNCGVTSNGRIYCIGGYDGSSIIPNVEAYDH
KM23-K13	FLYVFGGNNYDYKALFETEVYDRLRDVWYVSSNLNIPRRNNCGVTSNGRIYCIGGYDGSSIIPNVEAYDH
KM24-K13	FLYVFGGNNYDYKALFETEVYDRLRDVWYVSSNLNIPRRNNCGVTSNGRIYCIGGYDGSSIIPNVEAYDH
KM25-K13	FLYVFGGNNYDYKALFETEVYDRLRDVWYVSSNLNIPRRNNCGVTSNGRIYCIGGYDGSSIIPNVEAYDH
KM26-K13	FLYVFGGNNYDYKALFETEVYDRLRDVWYVSSNLNIPRRNNCGVTSNGRIYCIGGYDGSSIIPNVEAYDH
KM27-K13	FLYVFGGNNYDYKALFETEVYDRLRDVWYVSSNLNIPRRNNCGVTSNGRIYCIGGYDGSSIIPNVEAYDH
KM28-K13	FLYVFGGNNYDYKALFETEVYDRLRDVWYVSSNLNIPRRNNCGVTSNGRIYCIGGYDGSSIIPNVEAYDH
KM29-K13	FLYVFGGNNYDYKALFETEVYDRLRDVWYVSSNLNIPRRNNCGVTSNGRIYCIGGYDGSSIIPNVEAYDH
K1-K13	FLYVFGGNNYDYKALFETEVYDRLRDVWYVSSNLNIPRRNNCGVTSNGRIYCIGGYDGSSIIPNVEAYDH
3D7-K13	FLYVFGGNNYDYKALFETEVYDRLRDVWYVSSNLNIPRRNNCGVTSNGRIYCIGGYDGSSIIPNVEAYDH

S1 Fig. Sequencing alignment of K13 propeller region at amino acid position 435-690. The *P. falciparum* 3D7 and K1 strains are the artemisinin sensitive control. KM, Kota Marudu isolate and KB, Kalabakan isolate.

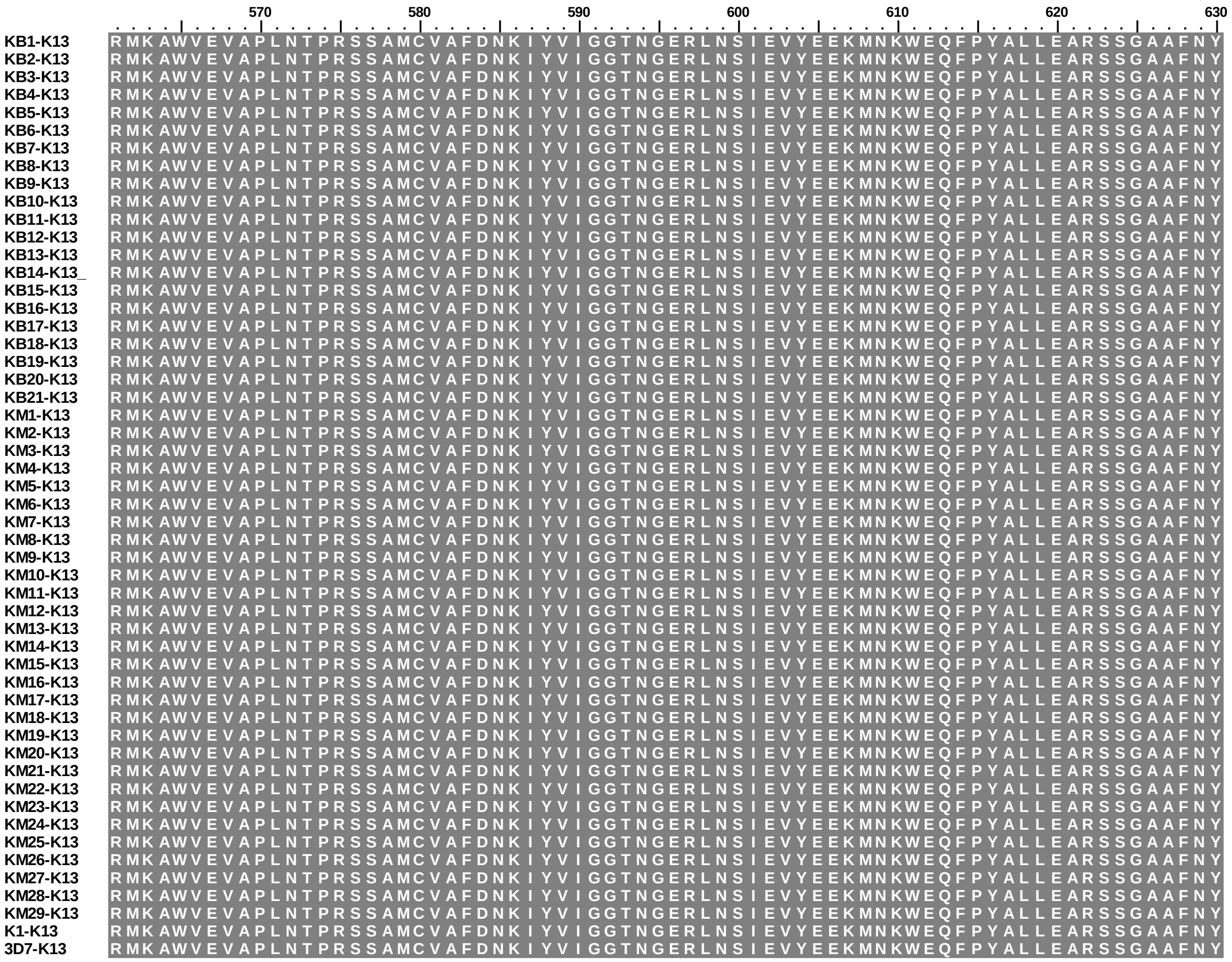

S1 Fig. Sequencing alignment of K13 propeller region at amino acid position 435-690. The *P. falciparum* 3D7 and K1 strains are the artemisinin sensitive control. KM, Kota Marudu isolate and KB, Kalabakan isolate.

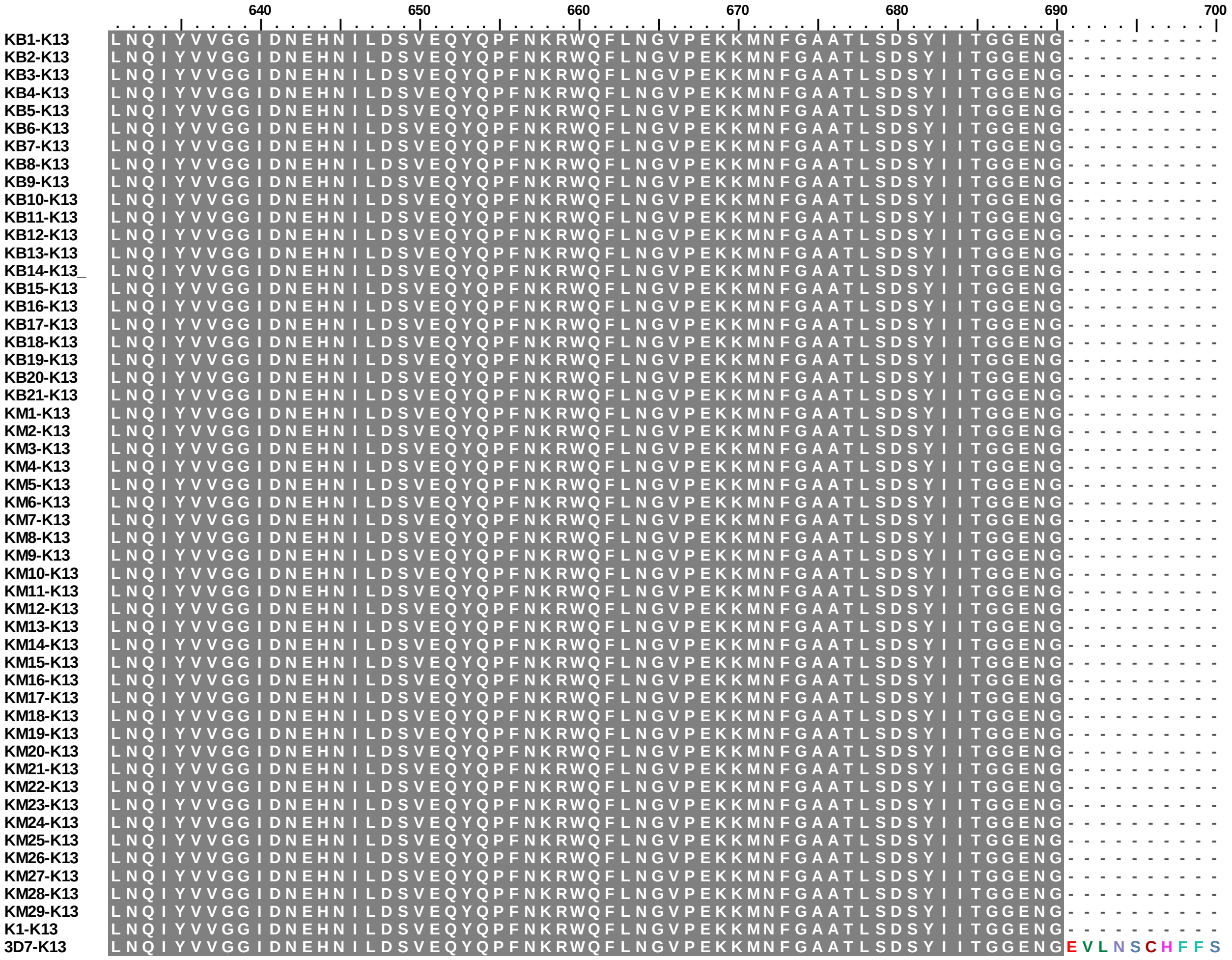

S1 Fig. Sequencing alignment of K13 propeller region at amino acid position 435-690. The *P. falciparum* 3D7 and K1 strains are the artemisinin sensitive control. KM, Kota Marudu isolate and KB, Kalabakan isolate.
