# Supplementary material for: Prevalence of Plasmodium falciparum Molecular Markers of Antimalarial Drug Resistance in a Residual Malaria Focus Area in Sabah, Malaysia
Source: PLoS One. 2016 Oct 27;11(10):e0165515. doi: 10.1371/journal.pone.0165515 (PMC5082862; doi:10.1371/journal.pone.0165515)
Supplement: S1 Table — (PDF) [file pone.0165515.s002.pdf]

S1 Table. Copy number of *pfmdr1* on the *P. falciparum* isolates.
Samples code: KB, Kalabakan; KM, Kota Marudu.

Sample name	Code	Copy number	OVERALL MEAN
**Control low copy no**	**3D7**	**1.04**	**0.97**
**Control low copy no**		**1.06**	
**Control low copy no**		**0.82**	
**Control low copy no**	**3D7**	**0.95**	**1.03**
**Control low copy no**		**1.08**	
**Control low copy no**		**1.07**	
**Control low copy no**	**3D7**	**1.05**	**1.01**
**Control low copy no**		**1.08**	
**Control low copy no**		**0.90**	
**Control high copy no**	**IC**	**2.59**	**2.52**
**Control high copy no**	**IC**	**2.35**	
**Control high copy no**	**IC**	**2.63**	
Sample	KB1	**1.01**	**1.00**
Sample	KB1	**1.06**	
Sample	KB1	**0.93**	
Sample	KB2	**1.03**	**1.05**
Sample	KB2	**1.07**	
Sample	KB2	**1.07**	
Sample	KB3	**0.93**	**0.88**
Sample	KB3	**0.75**	
Sample	KB3	**0.97**	
Sample	KB4	**0.83**	**0.99**
Sample	KB4	**1.14**	
Sample	KB4	**1.01**	
Sample	KB5	**0.76**	**0.91**
Sample	KB5	**1.07**	
Sample	KB5	**0.90**	
Sample	KB6	**0.91**	**1.01**
Sample	KB6	**1.36**	
Sample	KB6	**0.75**	
Sample	KB7	**0.92**	**0.96**
Sample	KB7	**1.04**	
Sample	KB7	**0.92**	
Sample	KB8	**0.93**	**0.83**
Sample	KB8	**0.88**	
Sample	KB8	**0.70**	
Sample	KB9	**1.01**	**1.04**
Sample	KB9	**1.11**	
Sample	KB9	**0.99**	
Sample	KB10	**1.06**	**1.08**
Sample	KB10	**0.91**	
Sample	KB10	**1.27**	
Sample	KB11	**0.82**	**0.90**
Sample	KB11	**0.89**	
Sample	KB11	**0.98**	
Sample	KB12	**1.60**	**1.14**
Sample	KB12	**0.70**	
Sample	KB12	**1.12**	
Sample	KB13	**1.04**	**1.03**
Sample	KB13	**1.04**	
Sample	KB13	**1.03**	
Sample	KB14	**1.15**	**1.35**
Sample	KB14	**No detection**	
Sample	KB14	**1.56**	
Sample	KB15	**1.07**	**1.13**
Sample	KB15	**1.13**	
Sample	KB15	**1.20**	
Sample	KB16	**1.12**	**1.11**
Sample	KB16	**1.04**	
Sample	KB16	**1.18**	
Sample	KM1	**1.39**	**1.43**
Sample	KM1	**1.48**	
Sample	KM1	**1.41**	
Sample	KM2	**0.90**	**0.94**

Sample	KM2	**0.91**	
Sample	KM2	**0.99**	
Sample	KM3	**1.15**	**1.10**
Sample	KM3	**1.09**	
Sample	KM3	**1.06**	
Sample	KM4	**1.36**	**1.35**
Sample	KM4	**1.07**	
Sample	KM4	**1.62**	
Sample	KM5	**1.07**	**1.20**
Sample	KM5	**1.21**	
Sample	KM5	**1.30**	
Sample	KM6	**1.22**	**1.20**
Sample	KM6	**1.31**	
Sample	KM6	**1.07**	
Sample	KM7	**1.19**	**1.28**
Sample	KM7	**1.27**	
Sample	KM7	**1.38**	
Sample	KM8	**1.24**	**1.20**
Sample	KM8	**0.99**	
Sample	KM8	**1.38**	
Sample	KM9	**1.25**	**1.36**
Sample	KM9	**1.30**	
Sample	KM9	**1.53**	
Sample	KM10	**No detection**	**0.84**
Sample	KM10	**0.88**	
Sample	KM10	**0.80**	
Sample	KM11	**1.23**	**1.17**
Sample	KM11	**1.16**	
Sample	KM11	**1.13**	
Sample	KM12	**1.16**	**1.10**
Sample	KM12	**0.97**	
Sample	KM12	**1.16**	
Sample	KM13	**0.95**	**1.05**
Sample	KM13	**1.15**	
Sample	KM13	**1.04**	
Sample	KM14	**1.67**	**1.40**
Sample	KM14	**1.38**	
Sample	KM14	**1.15**	
Sample	KM15	**1.15**	**1.34**
Sample	KM15	**1.19**	
Sample	KM15	**1.67**	
Sample	KM16	**1.29**	**1.27**
Sample	KM16	**1.24**	
Sample	KM16	**1.27**	
Sample	KM17	**0.90**	**0.89**
Sample	KM17	**0.97**	
Sample	KM17	**0.79**	
Sample	KM18	**0.92**	**0.89**
Sample	KM18	**0.86**	
Sample	KM18	**0.89**	
Sample	KM19	**0.90**	**1.00**
Sample	KM19	**1.02**	
Sample	KM19	**1.08**	
Sample	KM20	**0.96**	**0.96**
Sample	KM20	**1.08**	
Sample	KM20	**0.84**	
Sample	KM21	**1.05**	**1.04**
Sample	KM21	**1.01**	
Sample	KM21	**1.06**	
Sample	KM22	**1.07**	**0.95**
Sample	KM22	**1.07**	
Sample	KM22	**0.71**	
